# Improving Physical Health Monitoring in Lithium-Treated Patients Using a Cumulative Record Sheet

**DOI:** 10.1192/bjo.2025.10452

**Published:** 2025-06-20

**Authors:** Tyler Thomas, Christopher Ng, Daniel De Silva, Je Ssy Low

**Affiliations:** CTM UHB, Llantrisant, United Kingdom

## Abstract

**Aims:** Lithium is a widely prescribed mood stabiliser with proven efficacy in managing bipolar disorder, but its narrow therapeutic index necessitates regular physical health monitoring. Inadequate monitoring can increase risks for patients. Within the Taff Ely Community Mental Health Team (TECMHT), we identified a lack of a structured system to track and manage the necessary monitoring tests. This quality improvement project aimed to determine whether the implementation of a structured record sheet could improve adherence to the National Institute for Health and Care Excellence (NICE) physical health monitoring standards for lithium treatment in TECMHT.

**Methods:** A retrospective review of clinical records for 30 patients was conducted to establish baseline monitoring practices. A pre-implementation meeting with the nursing team highlighted existing challenges, including inconsistent monitoring and lack of centralised tracking. The intervention consisted of three components: (i) a standardised monitoring proforma, (ii) a formal clinic protocol, and (iii) a training session for clinic nurses. Following the implementation, prospective data for 20 patients attending the clinic during the 6-week monitoring period were analysed. Run charts were used to compare monitoring practices before and after the intervention, while Pareto charts identified areas for improvement and helped track compliance over time.

**Results:** Before the intervention, monitoring of BMI was not routinely performed, and calcium monitoring was suboptimal. Serum lithium level monitoring, however, showed good compliance with guidelines. After implementing the intervention, lithium level monitoring achieved 100% compliance with NICE guidelines, BMI monitoring was completed for 82% of patients, and calcium monitoring improved from 8% to 33%. A decline in compliance for urea and electrolytes and thyroid function monitoring was noted, which was attributed to initial confusion over the new system. This issue was addressed in a subsequent training session aiming to improve clarity and compliance.

**Conclusion:** A physical health monitoring form was successfully developed and integrated into the lithium monitoring clinic, improving access to monitoring data and ensuring better adherence to NICE guidelines. The form streamlined monitoring without requiring additional resources or labour. Following approval from senior health board management, the form is being rolled out across the health board. To assess its broader impact, further data analysis is recommended, and additional educational resources for clinic staff may be beneficial. This intervention demonstrates the value of structured systems in enhancing patient care in outpatient settings.

